# Changes of Nitric Oxide and Its Relationship with H_2_O_2_ and Ca^2+^ in Defense Interactions between Wheat and *Puccinia Triticina*

**DOI:** 10.1371/journal.pone.0132265

**Published:** 2015-07-17

**Authors:** Mei Qiao, Jiawei Sun, Na Liu, Tianjie Sun, Gang Liu, Shengfang Han, Chunyan Hou, Dongmei Wang

**Affiliations:** College of Life Science, Agricultural University of Hebei, Baoding, Hebei Province, China; Gyeongnam National University of Science and Technology, REPUBLIC OF KOREA

## Abstract

In this research, the wheat cultivar 'Lovrin 10' and *Puccinia triticina* races 165 and 260 were used to constitute compatible and incompatible combinations to investigate the relationship between NO and H_2_O_2_ and between NO and calcium (Ca^2+^) signaling in the cell defense process by pharmacological means. The specific fluorescent probe DAF-FM DA was coupled with confocal laser scanning microscopy and used to label intracellular nitric oxide (NO) and monitoring the real-time NO dynamics during the processes of wheat defense response triggered by *P*. *triticina* infection. The results showed that at 4 h after inoculation, weak green fluorescence was observed in the stomatal guard cells at the *P*. *triticina* infection site in the incompatible combination, which indicates a small amount of NO production. Twelve hours after inoculation, the fluorescence of NO in- cell adjacent to the stomata gradually intensified, and the NO fluorescent area also expanded continuously; the green fluorescence primarily occurred in the cells undergoing a hypersensitive response (HR) at 24–72 h after inoculation. For the compatible combination, however, a small amount of green fluorescence was observed in stomata where the pathogenic contact occurred at 4 h after inoculation, and fluorescence was not observed thereafter. Injections of the NO scavenger c-PTIO prior to inoculation postponed the onset of NO production to 48 h after inoculation and suppressed HR advancement. The injection of imidazole, a NADPH oxidase inhibitor, or EGTA, an extracellular calcium chelator, in the leaves prior to inoculation, delayed the onset of NO production in the incompatible combination and suppressed HR advancement. Combined with our previous results, it could be concluded that, Ca^2+^ and hydrogen peroxide (H_2_O_2_) are involved in upstream of NO production to induce the HR cell death during *P*. *triticina* infection, and Ca^2+^, NO and H_2_O_2_ are jointly involved in the signal transduction process of HR in the interaction system.

## Introduction

Plants have evolved numerous strategies to activate defensive responses against pathogen infection. In general, plants are capable of activating self-defense responses by quickly and efficiently producing a series of disease-resistant signal substances to defense the pathogen invasion [1]. Heo et al. have shown that plant defense responses include the following four stages that invariably involve a series of complex signal transduction processes: the recognition of pathogens by the host, the activation of host defense responses, the hypersensitive response (HR) form of cell death at the pathogen infection site, and the inhibition of pathogen growth [2]. Intensive studies have been performed on cell signaling, and studies on the crosstalk among signaling molecules are required for the continuous consummation of signal transduction models. Calcium (Ca^2+^), hydrogen peroxide (H_2_O_2_) and nitric oxide (NO) are important signal transduction molecules ubiquitous in plant and animal cells. As a common intracellular messenger, Ca^2+^ is widely involved in intracellular biochemical reactions and signal transduction processes. In the interactions between plants and pathogens, an increase in cytosolic Ca^2+^ ([Ca^2+^]_cyt_) concentrations is often the early-stage cellular response to pathogens infection, and this increase is also required to induce the HR form of cell death at the pathogen infection site [2–7]. Pathogenic infections lead to the rapid and significant accumulation of reactive oxygen species (ROS), a phenomenon known as oxidative burst. Maria et al. [8] showed that H_2_O_2_ concentrations were vital in enhancing the activity of antioxidant enzymes and resistance responses or inducing HR form of cell death at the pathogen infection site; in addition, the local burst or sustained release of ROS subsequent to pathogen infection initiates the complex multiple signal management network and leads to disease resistance in plants. The timing of ROS burst is also essential to hypersensitive cell death [9]. In rice, it was found that an increase in Ca^2+^ concentrations in mesophyll cells was necessary for the cells to produce ROS after pathogen infection [7]. It was also reported that alterations in Ca^2+^ concentrations in mesophyll cells not only initiated but also increased or sustained NO production. Lamotte et al. [10] demonstrated that by inducing the biosynthesis of NO in suspension cultured tobacco cells, NO synthesis was blocked immediately when a plasma membrane Ca^2+^ channel inhibitor was added; in addition, when Ca^2+^ concentrations were enhanced, NO could indirectly affect the activities of certain proteins (e.g., protein kinases, K^+^ or Cl^-^ channel proteins in guard cells) [11]. Recently, Ma et al. revealed that the increase of intracellular [Ca^2+^]_cyt_ is associated with cyclic nucleotide-gated channels (CNGCs) [12, 13], and the changes in [Ca^2+^]_cyt_ was closely related to NO production [14] in the process of plant-pathogen interaction. Since Ca^2+^ and NO can trigger HR form of cell death at the pathogen infection site [15, 16], the existence of a Ca^2+^-NO signaling pathway which occurs during the HR process is very likely. Moreover, orchestrated action by H_2_O_2_ and NO could trigger HR form of cell death at the pathogen infection site [17, 18]. Lin et al. found that the activity of nitrate reductase (NR) and the yield of NO is affected with H_2_O_2_ concentration in the process of cell death provoked by H_2_O_2_ in rice [19]. In a study on mung bean (*Phaseolus aureus*), Lum et al. found that exogenous H_2_O_2_ could induce production of NO in guard cells and that procedure probably be accomplished through the nitric oxide synthase (NOS) pathway [20].

Wheat (*Triticum aestivum* L.) leaf rust is a common and widespread disease caused by *P*. *triticina* infection, and its prevalence causes serious loss in wheat production. Investigations of the host resistance mechanism are the most economical, effective, sustainable and environmentally friendly method of controlling the disease [21], therefore attracted a lots of attention in the world. Our previous efforts in the molecular mechanism in wheat plants against leaf rust has demonstrated that the Ca^2+^-calmodulin (CaM) signal transduction pathway was involved in hypersensitive response induced by *P*. *triticina* infection [22–24]. In recent years, we used concentrated intercellular washing fluids (IWF) as an elicitor to stimulate wheat mesophyll protoplasts or wheat cell suspension cultures to simulate the interaction system of wheat and *P*. *triticina* and produced consistent findings [25–31]. Liu et al. demonstrated that an increase of [Ca^2+^]_cyt_ in response to the elicitor by the protoplasts of resistant wheat varieties was likely dependent on the influx of extracellular Ca^2+^, and a similar result was also observed in ultrastructure of wheat leaf infected by *P*. *triticina* [23]. Ren et al. studied the interaction system of an IWF-wheat cell suspension culture coupled with a pharmacological investigation and found that the H_2_O_2_ levels in the cell suspension culture increased after the IWF stimulation, which primarily originated from plasma membrane NADPH oxidase; in addition, they showed that there was a correlation between the elevation of [Ca^2+^]_cyt_ and H_2_O_2_ production [29]. In a study on the wheat and *P*. *triticina* interaction system, Qi found that an H_2_O_2_ burst occurred in HR cells induced by *P*. *triticina* infection and demonstrated that NADPH oxidase played an important role in the process [28]. Although the above described studies were based on two different systems and experimental methods, they reached very similar conclusion. Recently, Chen et al. found that NO mediated the HR form of cell death by using of the interaction system of wheat cell suspension culture and IWF [30]. Although Ca^2+^, H_2_O_2_ and NO play important roles as signaling molecules in plant defense reactions, the relationship among the three molecules is still in dispute.

In summary, Ca^2+^, NO and H_2_O_2_ are important signal transduction molecules in plant defence reaction, The relationship among Ca^2+^, H_2_O_2_ and NO or the crosstalk among the three are critical for understanding the mechanism of HR form of cell death induced by pathogen infection. *P*. *triticina* is a biotrophic parasite with high specificity, and HR is an important defense response to *P*. *triticina* in wheat plants. Investigations of the signal transduction relationship among Ca^2+^, H_2_O_2_ and NO in the interaction system between *P*. *triticina* and wheat can help determine the cellular and molecular mechanisms of HR. Hence, in this study, the wheat cultivar 'Lovrin 10' (L10) and *P*. *triticina* races 165 and 260 were used to create compatible and incompatible combinations. The specific fluorescent probe DAF-FM DA was used to label intracellular NO, and confocal laser scanning microscopy (CLSM) was used to observe the real-time NO dynamics during wheat defense response process triggered by *P*. *triticina* infection, and the relationship among NO, H_2_O_2_ and Ca^2+^ in the interaction was investigated using pharmacological methods to elucidate the mechanism of HR signal transduction induced by *P*. *triticina* in wheat.

## Materials and Methods

### Materials

The wheat cultivar ' Lovrin 10 ' (L10) and *P*. *triticina* physiological races 260 and 165 were used in this study, L10 and race 260 comprising the incompatible combination and L10 and race 165 comprising the compatible combination. The reproduction of *P*. *triticina* was conducted using the susceptible cultivar 'Zhengzhou 5389'.

### Cultivation and inoculation of wheat seedlings

Wheat seeds were planted in organic soil in 10-cm diameter pots, and grown in a greenhouse with a 16 h light/8 h dark cycle and a constant temperature of 25°C.

Seven-day old seedlings were used for *P*. *triticina* infection. According to Liu et al [23], fresh uredospore was suspended in water to a final concentration of 3×10^5^ spores.ml^-1^. Then, wheat seedlings were inoculated with the spore suspension by brushing it on the surface of the first leaf with a brush. Control plants were brushed with water only, referred to as mock inoculation. After inoculation, water was sprayed on the surface of the first leaf with a spray gun to simulate high humidity condition. Finally, inoculated seedlings were kept in a humid chamber for 16 h in the dark at 25°C to allow infection to occur, then, the seedlings were returned to greenhouse.

### Preparation of chemical reagent solutions

Sodium nitroprusside (SNP) was used as the NO donor at a final concentration of 2.5 mM. The working concentration of c-PTIO, an NO scavenger, was 200 μM. N-nitro-L-arginine methylester (L-NAME) was used as an analogue to the substrate of NOS at a working concentration of 100 μM; Na_2_WO_4_, an analogue to molybdenum, was used as an NR inhibitor at a working concentration of 100 μM. The working concentration of EGTA, the extracellular calcium chelator, was 5 mM, and that of imidazole, an inhibitor of NADPH oxidase, was 100 mM. The working concentration of DAF-FM DA was 50 μM. The working concentrations used in this study were determined by observing various concentrations of chemicals and their effects on the growth of wheat plants in the preliminary experiments.

### Detection of NO in wheat leaves

Subsequent to inoculation of the L10 leaf (7 days of age) with *P*. *triticina*, the leaf was sampled at different time points, with 1.0 cm segments collected from the upper, middle and lower parts of the leaf after the tip and base were discarded. The leaf samples were immersed in 1 mL of phosphate buffer (0.1 M, pH 7.2) and DAF-FM DA was added. A vacuum was then applied to the leaf samples using a syringe, to ensure the sampled tissues thoroughly soaked with the solution, and then following 2–3 times washes with distilled water. The leaf samples were observed under CLSM and photographed (at Ex/Em of 488 nm/526 nm), and cells with NO accumulation exhibited yellow-green fluorescence.

### Application of chemicals

The wheat seedlings were placed under fluorescent lights to prompt the opening of the stomata, and chemicals were injected into the leaves of wheat using the modified method by Deng [32].

### Observation of HR

At different time points after inoculation, the leaf was sampled, with 1.5 cm leaf segments collected from the lower, middle and bottom of each leaf after the tip and base of the leaf were discarded. The leaf samples were stained using the Rohringer fluorescent staining method [33]. The staining process was performed in a 27–29°C water bath, and the stained leaf samples were then observed under CLSM and photographed (at Ex/Em of 488 nm/526 nm for HR cells and Ex/Em of 358 nm/461 nm for the development of *P*. *triticina*). Under the microscope, the healthy host cells exhibited a pale green color, whereas the HR dead cells caused by *P*. *triticina* infection exhibited bright green fluorescence. Five leaves were sampled for each treatment, and 50 spots which infected by *P*. *triticina* were selected randomly for each treatment. The area of HR dead cells was measured for each of the selected spots, and the mean area was calculated for the 50 spots and plotted.

### NOS and NR activity assays

#### NOS activity assay

NOS activity was assayed with a nNOS kit (Nanjing Jiancheng Biological Co.) according to the manufacturer's recommendations. There are two main types of NOS that are included in the T-NOS (total NOS): cNOS (constitutive NOS) and iNOS (inducible NOS). The kit is capable of detecting the activity of iNOS and T-NOS. The enzymatic activity was represented using U mg^-1^ protein, where 1 unit is defined as the yield of brown substance produced per unit of protein per unit time when reacting with NO. The simulated inoculation was used as a control, and three replicates were set for each of the reactions.

#### NR activity assay

NR activity was assayed in accordance with routine laboratory assay methods. NR is a key enzyme used by plants to assimilate nitrogen, and it catalyzes the reduction of nitrate in plants to form nitrite (NO_3_ˉ + NADH + H^+^ → NO_2_ˉ + NAD^+^ + H_2_O), which then reacts with sulfanilic acid (or p-amino-benzenesulfonamide) and α-naphthylamine (or naphthyl ethylene diamine) under acidic conditions to quantitatively form a red azo compound that has a maximum absorption peak at 540 nm and can be determined using spectrophotometry. The activity of NR is represented by the amount of nitrite produced, which is generally designated as the amount of nitrite produced per unit time per unit weight of fresh leaf (μg g^-1^ h^-1^). The simulated inoculation was used as a control, and three replicates were set for each of the reactions.

## Results

### Development of a NO fluorescence labeling method for wheat leaves

**Fig 1:**
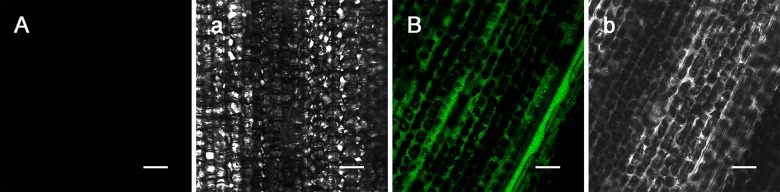
NO detection in leaves after PBS and SNP injections. Leaf injected with PBS (A). Leaf injected with SNP (B). Transmitted light photograph of A (a). Transmitted light photograph of B (b). (Bar = 50 μm).

First, the wheat leaves were injected with the NO donor SNP and then incubated with the fluorescent probe DAF-FM DA to test whether DAF-FM DA could be effectively penetrate into the wheat leaf to label NO. The DAF-FM DA incubation results (Fig 1) showed that after the DAF-FM DA incubation, no green fluorescent were observed under blue light excitation after PBS injection, whereas the leaf injected with SNP showed significant amounts of green fluorescence, indicating that the fluorescent probe DAF-FM DA in the test system could detect and accurately reflect the changes in NO levels in wheat leaves.

### NO changes in the interactions between wheat plants and *P*. *triticina*

**Fig 2:**
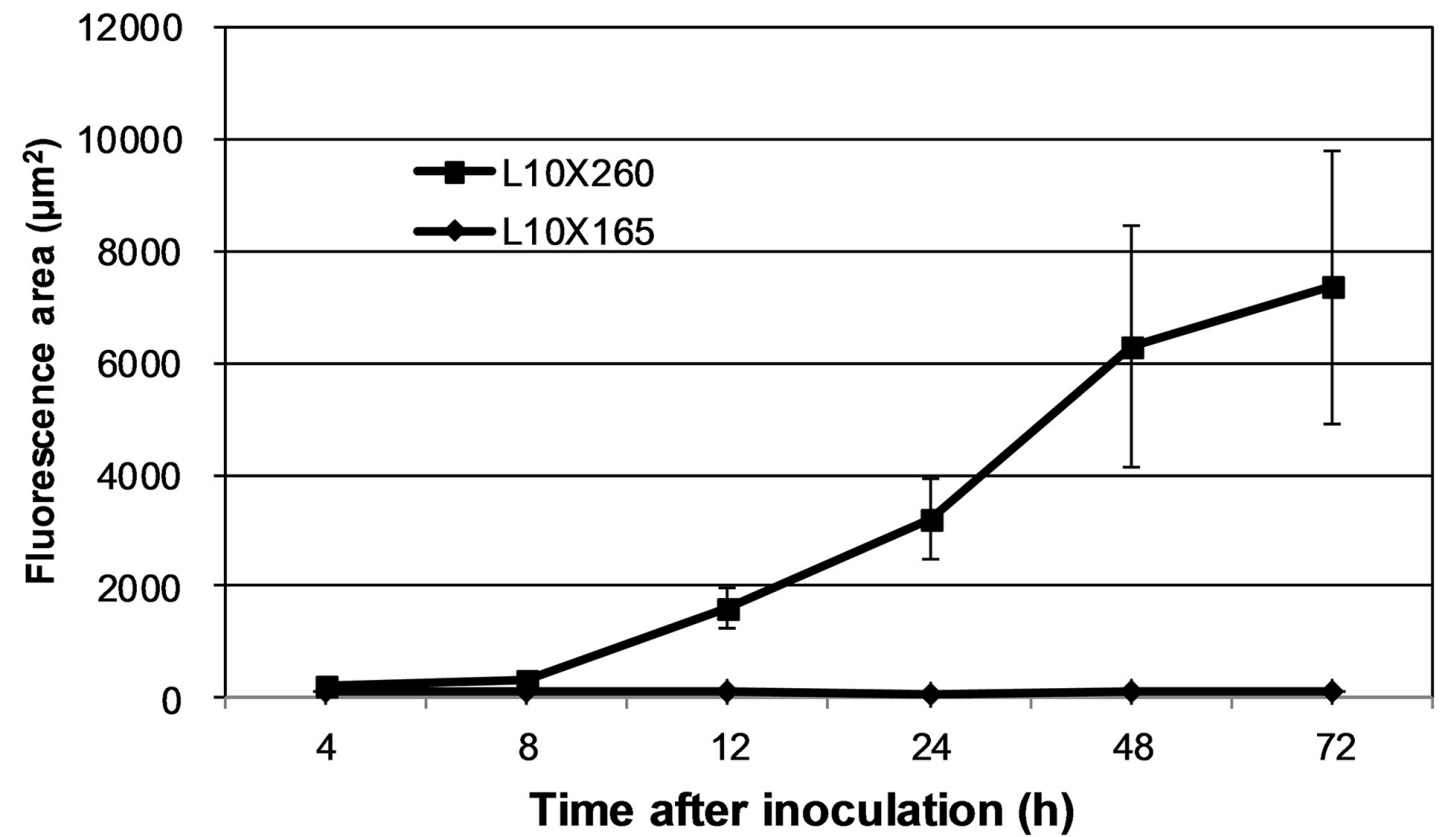
Changes of NO fluorescence area in different combinations. Each value was shown as mean ± SD (N = 3).

**Fig 3:**
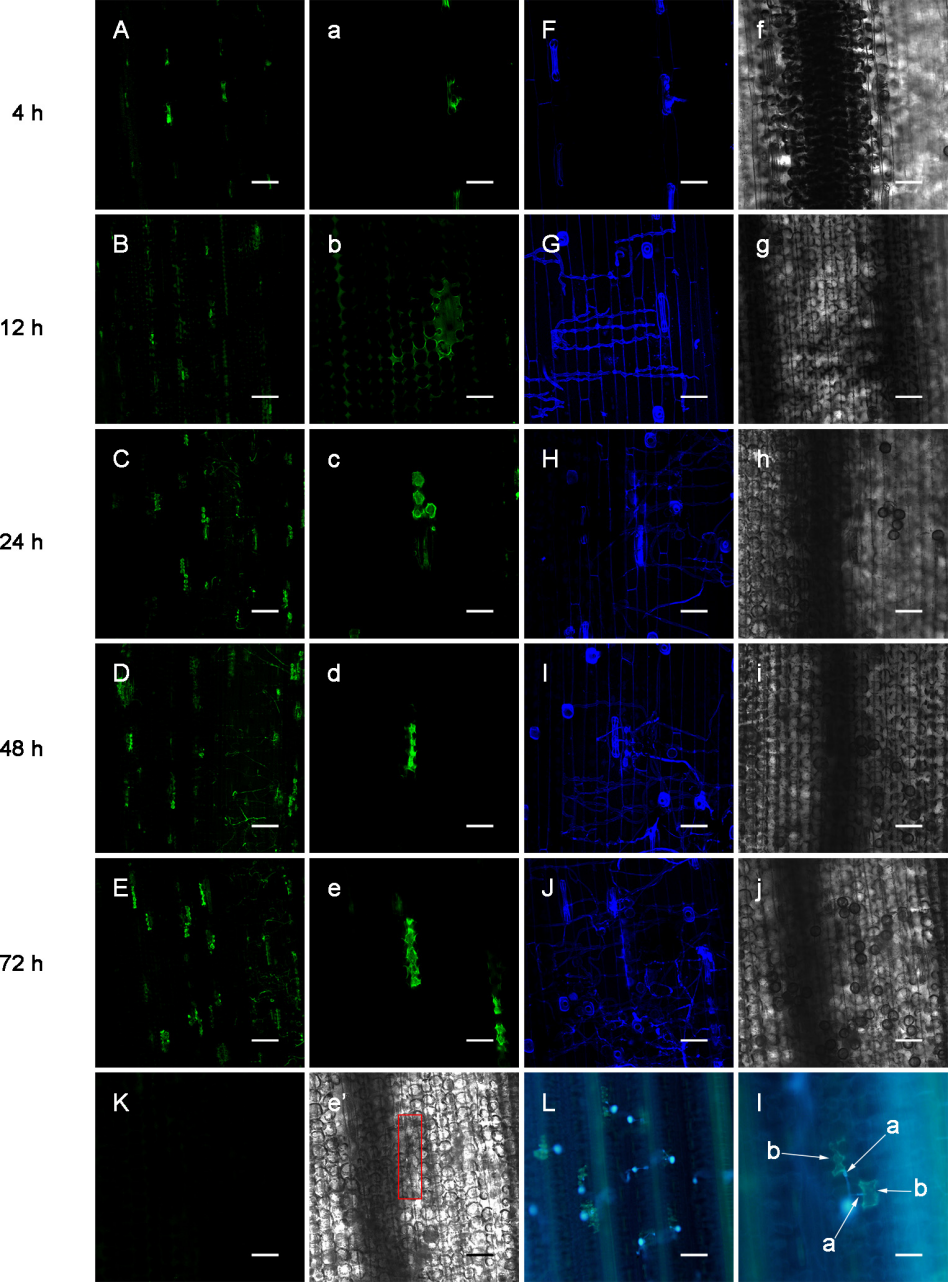
Changes of NO in L10 leaf after *P*. *triticina* infection. NO distributions in L10 leaves at 4, 12, 24, 48, 72 h after inoculation with *P*. *triticina* race 260 (A)—(E). Enlargements of A—E (a)—(e). Observations of *P*. *triticina* on the surfaces of a—e (F)—(J). Transmitted light photographs of F—J (f)—(j). At 72 h after inoculation with *P*. *triticina* race 165 (K). Transmitted light photograph of e (e'). Observations of HR and *P*. *triticina* at 72 h after inoculation with *P*. *triticina* race 260 (L). Enlargement of L, with a- haustorium mother cell; b- HR cells (l). (A—E, K: Bar = 150 μm; a—e, F—J, f—j, e': Bar = 50 μm; L: Bar = 122.5 μm; l: Bar = 61.25 μm)

The combinations used in this study are classic combinations that have been frequently used by our group. After inoculation with race 260, a limited number of L10 cells at the *P*. *triticina* infection site exhibited HR form of cell death at 16 or 24 h, and the area of cell death maximized at 72 or 96 h. However, in the compatible combination in which L10 was inoculated with *P*. *triticina* race 165, no HR death could be observed [28]. In this study, the leaves of the two combinations were sampled at 4–72 h after inoculation for NO labeling, and the results are shown in Figs 2 and 3. For the incompatible combination, weak green fluorescence could be observed at 4 h after inoculation in the guard cells of stomata in contact with *P*. *triticina*, indicating that trace amounts of NO were produced (Fig 3A and 3a). Over time, the NO fluorescence in the mesophyll cell around the infected site gradually intensified, and the fluorescent area expanded (Fig 3B and 3b). After 24–72 h post-inoculation, the green fluorescence concentrated primarily in the HR cells (Fig 3C and 3c; 3D and 3d; 3E and 3e), and the NO green fluorescent area maximized at 72 h (Fig 2), which is consistent with our previous observations for this combination, wherein the HR area reached the maximum at 72 h or 96 h after inoculation. In addition, the HR staining at 72 h after inoculation showed that each haustorium mother cell generally corresponded to one HR cell and that there were 1–2 haustorial mother cells at each of the infection spots (Fig 3L and 3l). For the compatible combination, the extremely weak NO fluorescence was only observed at the stomata of the infection site at 4 h after inoculation, and NO fluorescence was not observed hereafter (Fig 3K, 72 h after inoculation). These results showed that NO production in the interaction between wheat plants and *P*. *triticina* varied widely among different combinations, with the incompatible combination producing NO in the early stage after inoculation and NO yields increasing over time, and limited NO production observed in HR cells, suggesting that NO may be associated with HR defense in wheat plants induced by *P*. *triticina*.

### Changes in NR and NOS activities in the interactions between wheat and *P*. *triticina*

NO is a gas molecule that occurs in many living organisms [34, 35], and it is primarily synthesized by the NOS and NR pathways [36, 37]. However, the gene sequence of nNOS in plants has not been determined, although numerous studies found that when treating plant samples with L-NAME, NO production was effectively inhibited, which suggests the occurrence of NOS analogues in plants.

**Fig 4:**
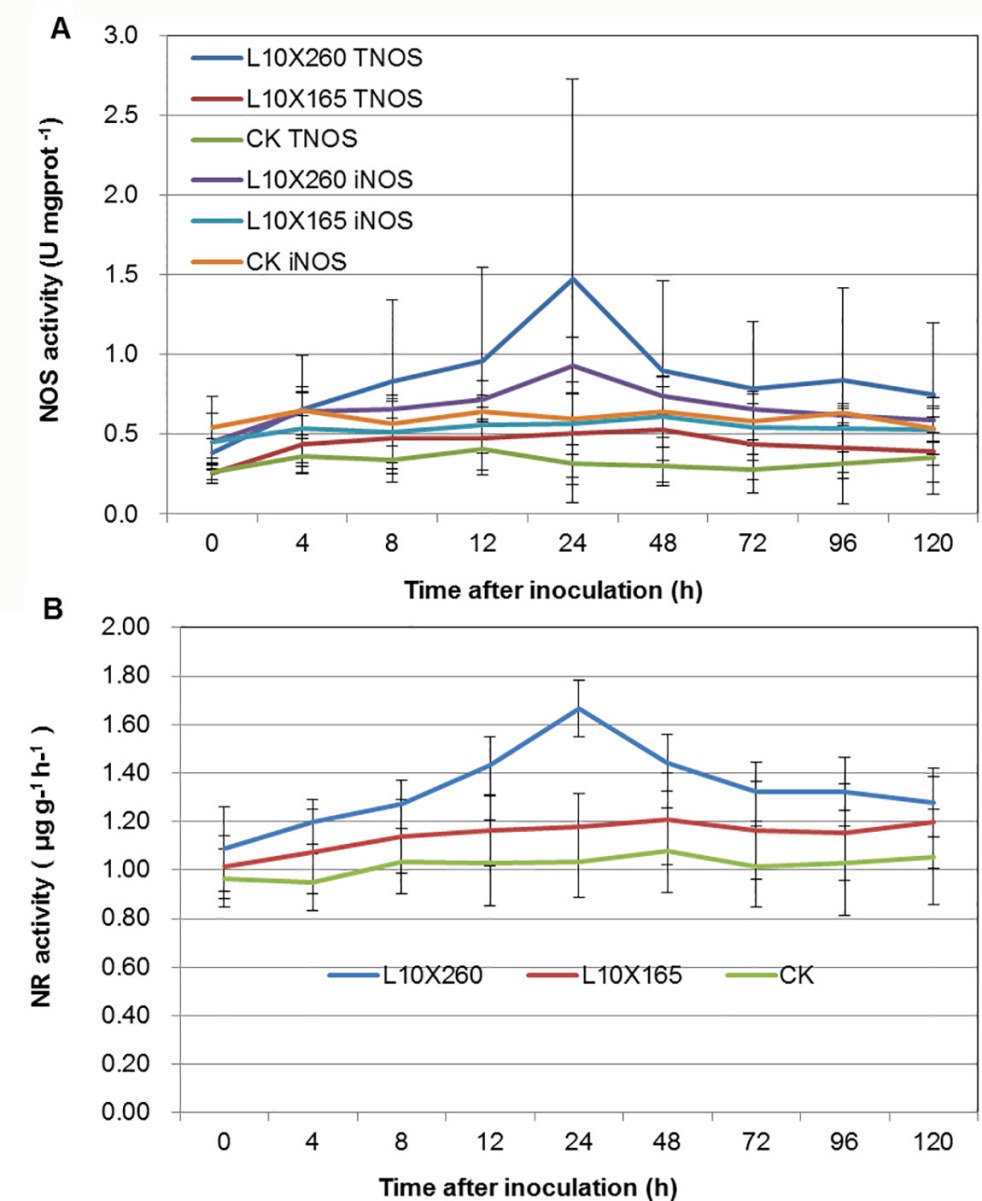
Changes of NOS and NR activities in different combinations. NOS activity (A). Results were expressed as unit activity per milligram protein (U mgprot^-1^). NR activity (B). Each value was shown as mean ± SD (N = 3).

The activities of NOS and NR in the leaf at different time points after inoculation were assayed in this study, and the results are shown in Fig 4. For the incompatible combination, the T-NOS activity and iNOS activity increased at 0–24 h after inoculation, and the activity of T-NOS was always higher than that of iNOS, but the activities of NOS and NR decreased steadily after 24 h, with higher T-NOS activity in the incompatible combination than in the compatible combination. However, for the compatible combination, the change in enzyme activity was insignificant, and a similar overall trend to the control was observed (Fig 4A). The trends of change in NR activity were also similar to those of NOS activity in both of the combinations, with the NR activity in the incompatible combination increasing rapidly at 4–24 h after inoculation, peaking at 24 h and then decreasing, while compatible combination showing lower levels than the incompatible combination. For the compatible combination, changes in NR activity were insignificant and similar to that of the control group (Fig 4B). These results demonstrated that in the incompatible combination between wheat and *P*. *triticina*, NOS and NR-mediated enzymatic reaction pathways might provide important contributions to the NO production.

### Effect of the injected chemicals on NO and HR in the interactions between wheat and *P*. *triticina*

#### Influence of the pre-injection of c-PTIO on NO and HR in the interactions between wheat and *P*. *triticina*

**Fig 5:**
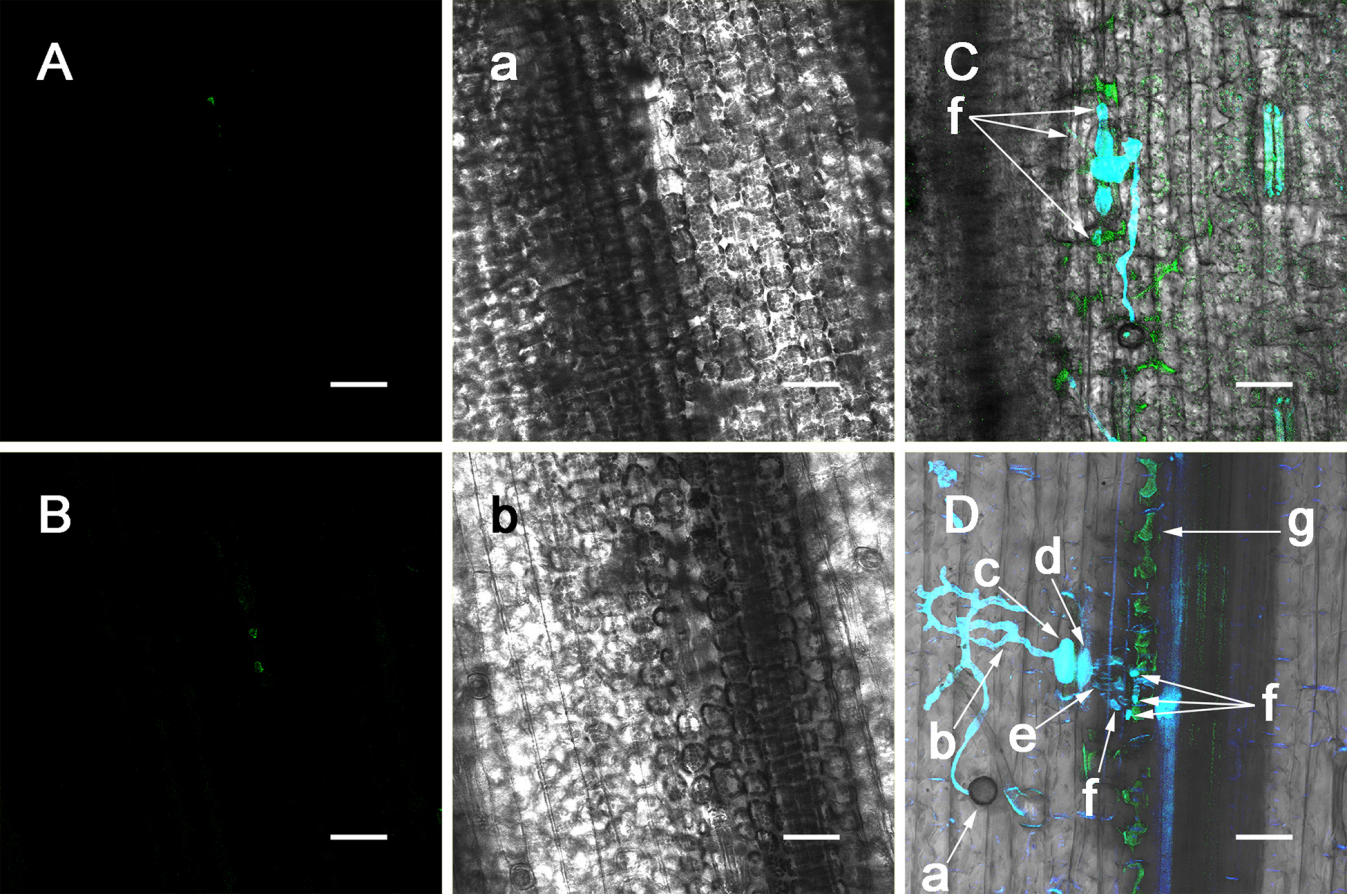
Effect of c-PTIO pre-injection on NO and HR. At 48 h after inoculation (A). At 72 h after inoculation (B). Development of HR and *P*. *triticina* at 96 h after inoculation (C). Development of HR and *P*. *triticina* at 120 h after inoculation: a- spores; b- germ tube; c- appressorium; d- substomatal vesicles; e- intercellular hyphae; f- haustorium mother cell; g- HR dead cell (D). Transmitted light photograph of A (a); Transmitted light photograph of B (b). (A, B, a, b: Bar = 50 μm; C, D: Bar = 37.5 μm)

**Fig 6:**
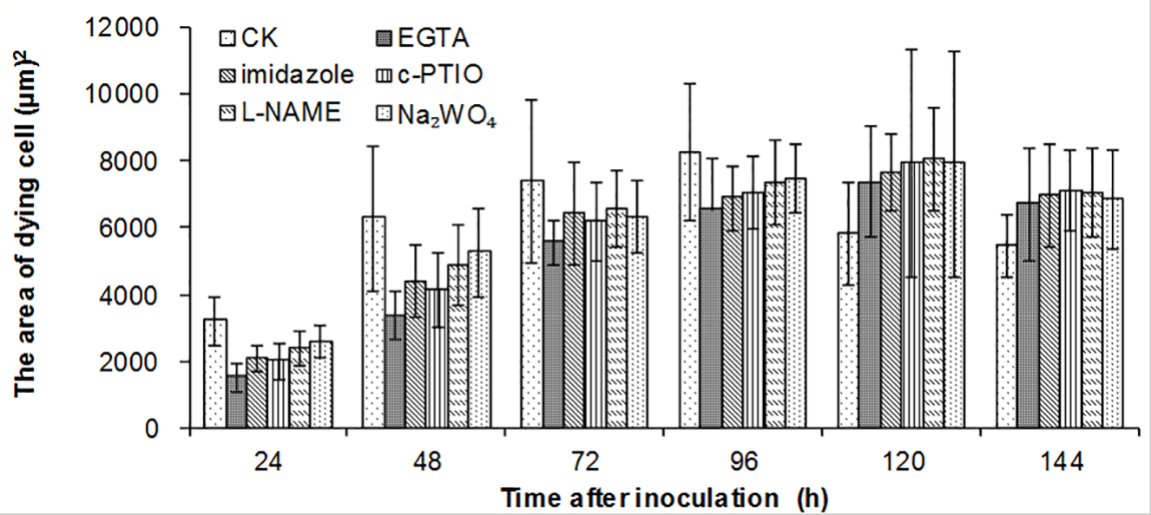
Effect of different chemical pre-injections on HR. Each value was shown as mean ± SD (N = 3)

As it is showed above, in the interactions between wheat plants and *P*. *triticina*, significant amounts of NO were produced primarily in the HR cells, suggesting that NO may be closely associated with the occurrence of HR. To confirm this hypothesis, the following pharmacological experiments were conducted, with c-PTIO, a scavenger of NO, injected into the L10 leaf prior to inoculation with the race 260, and the leaf samples were collected at different time points after inoculation to determine NO production and to observe the HR; the results are shown in Fig 5. After c-PTIO injection, no green fluorescence could be observed at 4–48 h after inoculation (Fig 5A- at 48 h after inoculation), and a faint NO fluorescence was observed in HR dead cells at 72 h after inoculation (Fig 5B). In addition, the development of HR cells (Figs 5, 5C, 5D and 6) and *P*. *triticina* at each of the time points was investigated (Fig 5C and 5D). Compared with the results we discussed above, the c-PTIO scavenged NO that was previously generated in the leaf, therefore, the rate of HR advancement was decreased, and the HR area peaked at 120 h after inoculation (Fig 6), which was 24–48 h later than control, although the number of haustorium mother cells at each of the infection spots increased to 3–4. These results indicated that NO likely participates the HR process in the wheat plant in response to *P*. *triticina* infection and that NO scavenging suppressed HR advancement, which was more favorable for the growth and development of *P*. *triticina*. However, the efficacy of c-PTIO reduced overtime, and NO subsequently accumulated and rapidly triggered HR in the host cells, with the HR area peaking at 120 h after inoculation (Fig 6).

#### Influence of the pre-injection of L-NAME or Na_2_WO_4_ on NO and HR in the interactions between wheat plants and *P*. *triticina*

In results we described above, we showed that in the incompatible combination between wheat plants and *P*. *triticina*, NOS and NR activities increased over time after inoculation and presumably contributed to NO production. To determine the enzymatic pathway that played the primary role in NO-mediated HR, inhibitors of NOS and NR were applied in the experiments.

**Fig 7:**
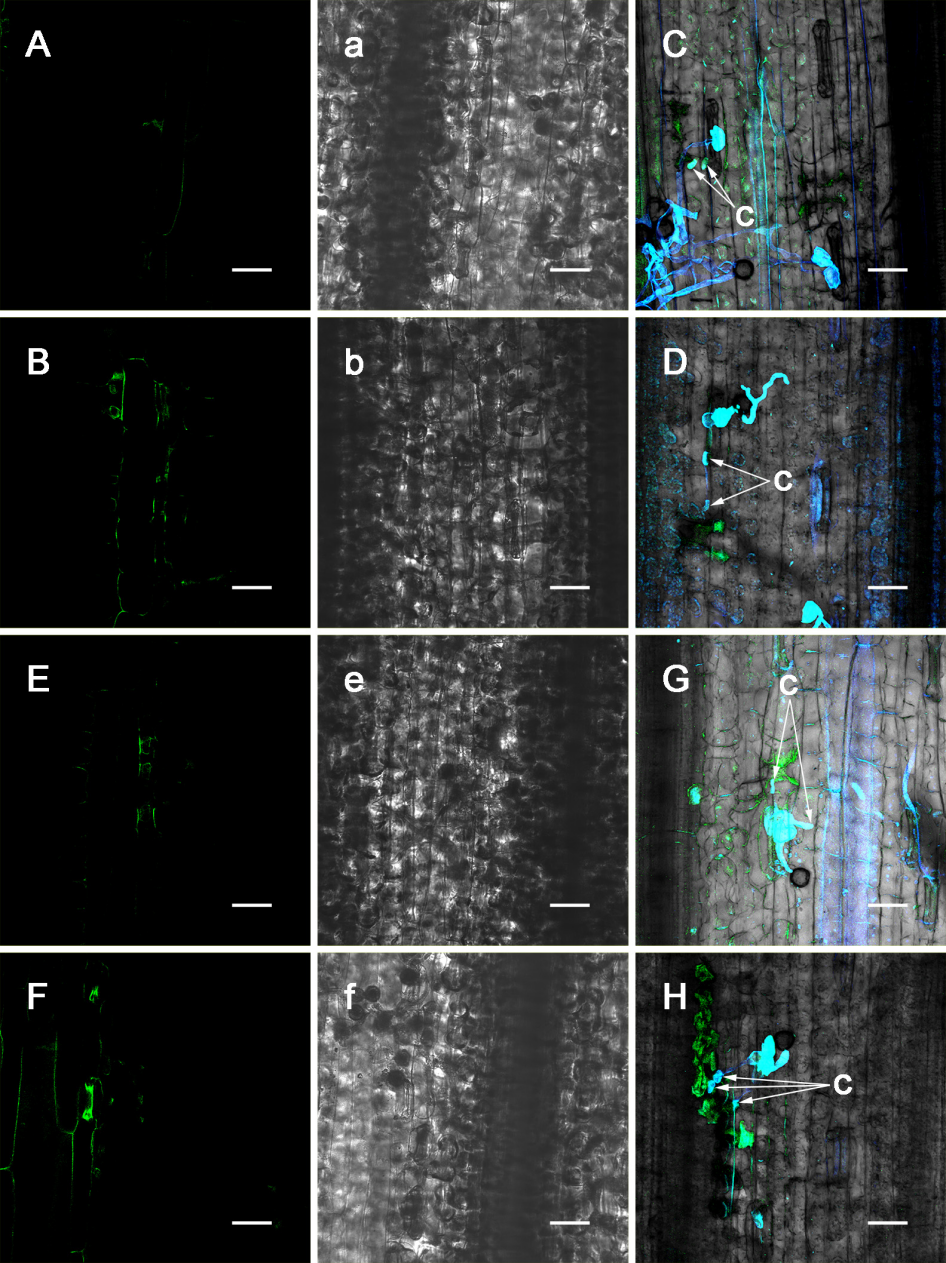
Effect of L-NAME or Na_2_WO_4_ pre-injection on NO and HR. At 48 h after inoculation (L-NAME pre-injection) (A). At 72 h after inoculation (L-NAME pre-injection) (B). Development of HR and *P*. *triticina* at 96 h after inoculation (L-NAME pre-injection) (C). Development of HR and *P*. *triticina* at 120 h after inoculation (L-NAME pre-injection) (D). At 48 h after inoculation (Na_2_WO_4_ pre-injection) (E). At 72 h after inoculation (Na_2_WO_4_ pre-injection) (F). Development of HR and *P*. *triticina* at 96 h after inoculation (Na_2_WO_4_ pre-injection) (G). Development of HR and *P*. *triticina* at 120 h after inoculation (Na_2_WO_4_ pre-injection) (H). Transmitted light photograph of A (a). Transmitted light photograph of B (b). Transmitted light photograph of E (e). Transmitted light photograph of F (f). haustorium mother cell (c). (A, B, a, b, E, F, e, f: Bar = 50 μm; C, D, G, H: Bar = 37.5 μm)

L-NAME and Na_2_WO_4_ are inhibitors of NOS and NR, respectively. Prior to the inoculation with race 260, L-NAME and Na_2_WO_4_ were injected into the L10 leaves, and leaf samples were collected at different time points after inoculation to determine NO production and to observe HR; the results are shown in Fig 7. After injection with L-NAME or Na_2_WO_4_, the NO behavior at 4–48 h after inoculation was similar to that with c-PTIO pre-injections but with a slightly higher NO fluorescence intensity (Fig 7A and 7E- at 48 h after inoculation), and significant accumulations of NO were observed in the HR cells at 72 h after inoculation (Fig 7B and 7F). In addition, the development of HR cells and *P*. *triticina* at each of the time points was investigated (Figs 6, 7C, 7D, 7G and 7H). The results showed that regardless of whether the NOS inhibitor L-NAME or NR inhibitor Na_2_WO_4_ was injected, the treatment suppressed HR advancement (Fig 6), which was beneficial to the growth and development of *P*. *triticina* (Fig 7C, 7D, 7G and 7H). Because 2–3 haustorium mother cells were usually found at each of the infection spots, which was a higher number than the control, the NOS and NR pathways likely participated simultaneously in the NO-mediated HR process.

#### Influence of pre-injection of EGTA on NO and HR in the interactions between wheat plants and *P*. *triticina*

**Fig 8:**
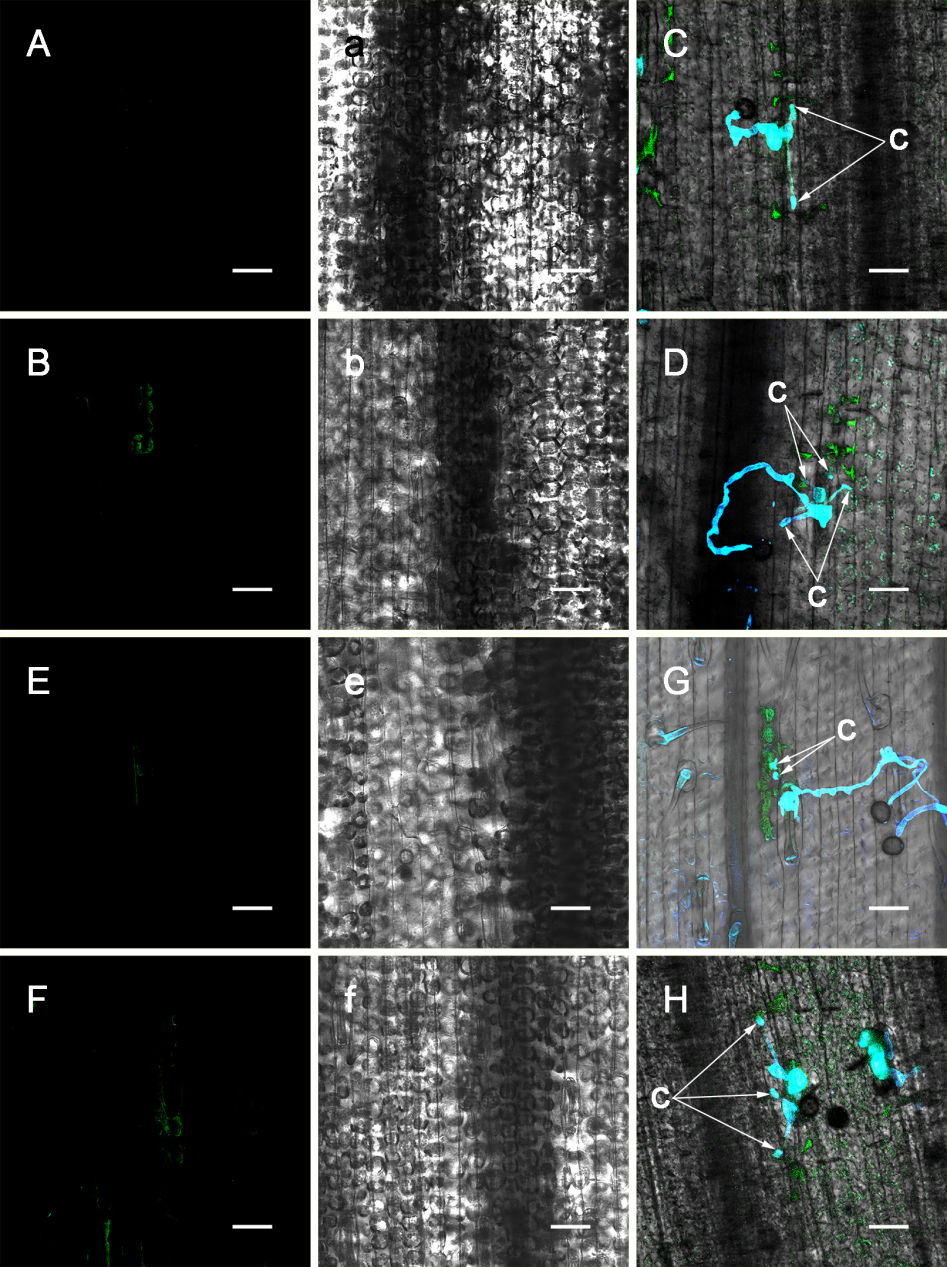
Effect of EGTA or imidazole pre-injection on NO and HR. At 48 h after inoculation (EGTA pre-injection) (A). At 72 h after inoculation (EGTA pre-injection) (B). Development of HR and *P*. *triticina* at 96 h after inoculation (EGTA pre-injection) (C). Development of HR and *P*. *triticina* at 120 h after inoculation (EGTA pre-injection) (D). At 48 h after inoculation (imidazole pre-injection) (E). At 72 h after inoculation (imidazole pre-injection) (F). Development of HR and *P*. *triticina* at 96 h after inoculation (imidazole pre-injection) (G). Development of HR and *P*. *triticina* at 120 h after inoculation (imidazole pre-injection) (H). Transmitted light photograph of A (a). Transmitted light photograph of B (b). Transmitted light photograph of E (e). Transmitted light photograph of F (f). haustorium mother cell (c). (A, B, a, b, E, F, e, f: Bar = 50 μm; C, D, G, H: Bar = 37.5 μm)

As previously mentioned, we have performed various studies upon the changes and roles of Ca^2+^ in the early stages of wheat plant and *P*. *triticina* interactions and revealed that as a signal molecule, Ca^2+^ is essential to HR induced by *P*. *triticina* infection [38] and that the increased [Ca^2+^]_cyt_ primarily resulted from an extracellular calcium influx [23]. To detect the effect of [Ca^2+^]_cyt_ on cytosolic NO, EGTA, an extracellular calcium chelator, was employed in this study. After the pre-injection of EGTA into L10 leaves, NO green fluorescence was not observed at 4–48 h after inoculation with race 260, and weak NO fluorescence was observed at 72 h after inoculation (Fig 8A and 8B). In addition, the development of HR cells and *P*. *triticina* at each of the time points was investigated (Figs 6, 8C and 8D), and the results were similar to those with c-PTIO pre-injections. Thus, we speculated that EGTA chelated and blocked the influx of extracellular calcium, which affected the infection-induced NO production and lead to a suppression of NO-mediated HR and postponed the maximum HR area to 120 h after inoculation (Fig 6). The preliminary results shown here indicate that NO production in the incompatible combination between L10 and *P*. *triticina* originated from the influx of extracellular calcium and show that calcium operates upstream of NO in the infection-induced HR process.

### Influence of pre-injection of imidazole on NO and HR in the interactions between wheat plants and *P*. *triticina*

Qi from our group found that in the interactions between wheat and *P*. *triticina*, an H_2_O_2_ burst occurred in the infection-induced HR, confirming that H_2_O_2_ generation was closely related to NADPH oxidase [28]. To further test the influence of H_2_O_2_ on cytosolic NO, imidazole, an NADPH oxidase inhibitor, was used in this study. After the pre-injection of imidazole prior to inoculation, a faint NO green fluorescence was observed in the cells at the infection site of *P*. *triticina* at 48 h after inoculation (Fig 8E- 48 h after inoculation), and a small amount of NO green fluorescence was concentrated in the HR cells at 72 h after inoculation (Fig 8F). By observing the development of *P*. *triticina* and HR cells, the pre-injection of imidazole was found to suppress HR advancement in the host cells (Fig 6), and the number of haustorium mother cells also increased to 3–4 (Fig 8G and 8H). These results indicated that imidazole inhibited NADPH oxidase activity, thereby affecting the generation of H_2_O_2_, inhibiting the infection-induced NO production and eventually suppressing the advancement of NO-mediated HR. It was speculated that in the process of *P*. *triticina* infection-induced HR, H_2_O_2_ operates upstream of NO.

### Influence of chemical pre-injections on the growth of *P*. *triticina*

**Fig 9:**
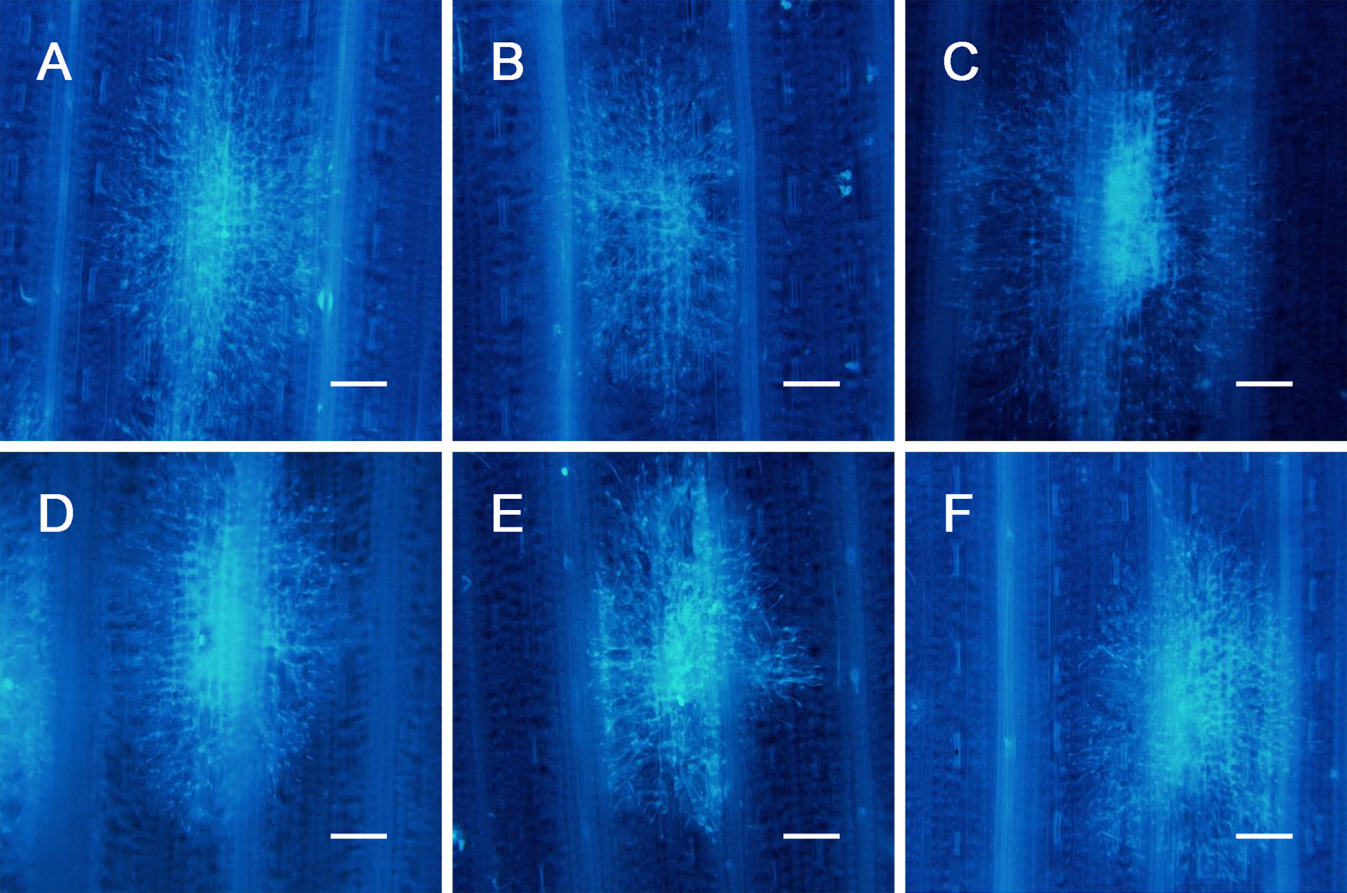
Hyphae expansion at 120 h after pre-injection of different chemicals. PBS buffer (A). c-PTIO (B). EGTA(C). imidazole (D). L-NAME (E). Na_2_WO_4_ (F). (A-F: Bar = 122.5 μm)

**Fig 10:**
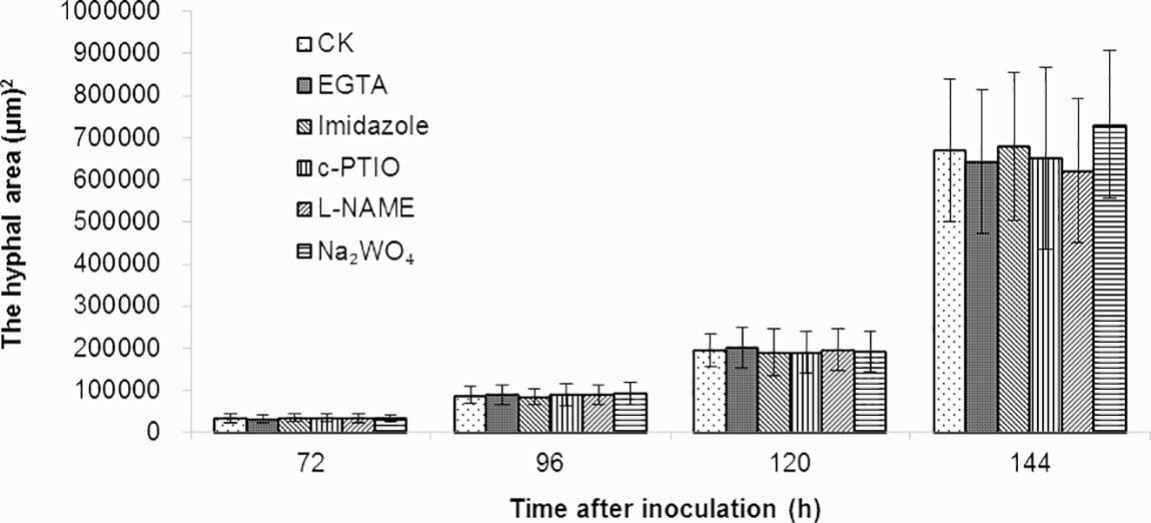
Effect of different chemicals on hyphae expansion area in compatible combinations. Each value was shown as mean ± SD (N = 3)

To examine the influence of pre-injected chemicals, i.e., c-PTIO, L-NAME, Na_2_WO_4_, EGTA and imidazole, on the growth of *P*. *triticina*, the chemicals were first injected in the leaves of ‘Zhengzhou 5389’, which were then inoculated with race 260, forming a compatible combination; PBS was injected into leaves that were then inoculated with race 260, with this treatment considered the control. The leaves were sampled at different time points after inoculation, and the hyphae expansion area was measured under a fluorescence microscope; the results are shown in Figs 9 and 10. Compared with the control, the hyphae expansion of the chemical pre-injection treatments at 72–144 h after inoculation showed no significant differences, and similar findings were observed at each of the time points. Therefore, it is concluded that the chemical concentrations used in the current experiment exerted no influence on the growth of *P*. *triticina*.

## Discussion

### Function and possible sources of NO in the interactions between wheat plants and *P*. *triticina*

The process of the plant resist stress is the one which involves various signaling molecules participating cellular signal transduction. Under abiotic stress, plants resist pathogen infections using NO as a signaling molecule; therefore, the source and production mechanism of NO are important issues. NO production can be achieved through enzymatic or non-enzymatic reaction pathways, and most related studies are focused on the source of enzymatic reaction pathways. Currently, three enzymes are considered to be associated in the primary production of NO in plants: NR, NOS and xanthine oxidoreductase (XOR). In mammalian cells, three forms of NOS, eNOS (endothelial NOS), nNOS (neuronal NOS) and iNOS, have been identified [35]. However, in an analysis of the whole-genome sequencing of Arabidopsis and rice, no gene or protein has been found to be homologous with animal NOS. Immunohistochemical techniques and pharmacological experiments (mainly using substances such as L-NAME that inhibit animal NOS activity) have been used to identify substances with activities similar to NOS occur in plants [39–41]. Another route of NO production in plants is its synthesis by NR [42–44]. As an analogue of molybdenum, Na_2_WO_4_ can decrease the activity of NR through pretreatment of the cells; therefore, it has frequently been used as an NR inhibitor [45]. In an Arabidopsis cell suspension culture, Na_2_WO_4_ has been found to reduce NO production increased by cladosporin [46]. This study found that NO production occurred in incompatible combination between wheat plants and *P*. *triticina* (Figs 2 and 3). To determine the enzymatic pathway that play a primary role in the NO production process, the three inhibitors were used, and the effect of the NOS inhibitor L-NAME or NR inhibitor Na_2_WO_4_ was similar to that of c-PTIO, indicating that in incompatible combination, the NO was primarily formed through the two NR and NOS enzymatic reaction pathways. Observations of HR cells in the pharmacological experiments indicated that the chemicals inhibited NO production in the early stages of the interaction and suppressed HR advancement (Fig 6), suggesting that in incompatible combination between wheat plants and *P*. *triticina*, NO acts as an important signaling molecule and mediates the occurrence of HR. An assessment of the growth of *P*. *triticina* showed that the suppressed advancement of HR promoted the secondary growth and development of *P*. *triticina* (Figs 5 and 7), which exhibited a greater number of haustorium mother cells than in the control. This result is mainly caused by *P*. *triticina* manifesting biotrophic parasite characteristics with high specificity; thus, the occurrence of HR in the host cells greatly blocks the nutrient sources for the invading pathogen and restricts the development of *P*. *triticina*. Once HR advancement is suppressed, it is equivalent to providing the pathogen with additional nutrients, which is beneficial for the development of *P*. *triticina*.

### Possible relationship between NO and Ca^2+^ signals in the interactions between wheat plants and *P*. *triticina*

Ca^2+^ and NO are common signaling molecules in plants and help regulate plant growth and development at various stages as well as plant responses to biotic and abiotic stresses. Ca^2+^ is a second messenger which plays a critical role in plant growth and development [47]. In addition, Ca^2+^ is involved in regulating various cellular processes, such as switching stomata on and off [48]. Different plant cells are affected by numerous factors, such as mechanical stimulation, cold stress, water stress, salt stress, oxidative stress and elicitor stimulation; the initial reaction is almost always a change in [Ca^2+^]_cyt_, and the signal is transduced and amplified through calcium-binding proteins such as calmodulin, calcium-dependent protein kinase and calcineurin [49].

NO also has important regulatory functions in the interactions between plants and pathogens. Particularly, the HR form of cell death at the pathogen infection site and gene expression triggered by NO acting as a signaling substance has become a focus in researches related [50]. Among plant signaling molecules, changes in the concentration of Ca^2+^ regulate the synthesis of NO [51], with Ca^2+^ acting as either an elicitor of NO signals or transmitter of NO signals and interacting with NO. It has also been reported that induced NO production in tobacco originates from calcium signals and that NO production induces the continuous increase of Ca^2+^ concentration [52]. Ma et al. [53] found that in fungal triggered immune signaling pathways in Arabidopsis, NO, H_2_O_2_ and calcium-dependent protein kinase (CDPK) were downstream of early-stage calcium signaling; however, the relationship between [Ca^2+^]_cyt_ and NO signals in the signal transduction pathway of the host response to pathogen infections is still inconclusive.

Through studies on the changes in extracellular calcium and calmodulin concentrations and pharmacological studies on the chemicals that affect calcium metabolism and calcium channels [38], the subcellular localization of Ca^2+^ in mesophyll cells and the changes of [Ca^2+^]_cyt_ in the elicitor-protoplast system, our previous study has shown that the calcium messenger system mediates the interactions between wheat plants and *P*. *triticina* and that Ca^2+^ influx is essential for the HR defensive response to *P*. *triticina* infection [23, 24]. To further investigate whether Ca^2+^ has any influence on the formation of NO in these interactions, EGTA was used in this study. The results showed that NO green fluorescent was not observed at the infection site following the injection of EGTA at 4–48 h after inoculation, NO fluorescence was mostly concentrated in HR cells at 72 h after inoculation (Fig 8B), HR advancement was suppressed compared to that of the control (Fig 6), at the same time the number of haustorium mother cells was higher than that of the control. Real-time changes in [Ca^2+^]_cyt_ and NO in the interactions of an elicitor-cell suspension culture have been monitored and showed that the application of EGTA greatly reduced [Ca^2+^]_cyt_ in the L10 cell suspension culture induced by elicitor to levels similar to that of the control [31], which indicates the elicitor-induced [Ca^2+^]_cyt_ increase in the cell suspension culture primarily originated from the influx of extracellular Ca^2+^. Combining with results we stated in this research, we speculate that in the incompatible combination between wheat plants and *P*. *triticina*, Ca^2+^ influx induced the production of NO, which mediated the occurrence of HR; therefore, in the signal transduction pathway of HR induced by *P*. *triticina* infection, Ca^2+^ operates upstream of NO. Whether NO provides feedback regulation on the concentrations of intracellular Ca^2+^ requires further study.

### Possible relationship between NO and H_2_O_2_ in interactions between wheat plants and *P*. *triticina*

When plant responses to biotic and abiotic stresses, NO and H_2_O_2_ are important signaling molecules that regulate plant secondary metabolism and the activity of protein enzymes [54–56]. NO and H_2_O_2_ signals can both be transported across membranes and are interrelated; however, the pathway relationship between the two is unknown. Several studies on stomatal movement of guard cells have suggested that NO production likely plays a role downstream of H_2_O_2_; for example, in the stomatal closure of guard cells triggered by darkness in ultraviolet-B radiation (UV-B) and abscisic acid (ABA) treatments, NO synthesis relied on the H_2_O_2_ generated in the guard cells [57, 58]. However, NO has been reported to regulate H_2_O_2_ generation and to induce the expression of relevant defense-related downstream genes, and in broad bean guard cells, NO can regulate the accumulation of H_2_O_2_ [59]. Liu et al. [60] reported that in the regulation process of stomatal movement by ethylene, H_2_O_2_ was likely at upstream of NO and regulated the formation of NO by regulating NR activity, which ultimately leads to stomatal closure in *Arabidopsis thaliana*.

Our previous results have demonstrated that H_2_O_2_ can mediate HR in host cells in the incompatible combination between wheat plants and *P*. *triticina*. Pharmacological analysis indicated that in the interaction system, the influx of Ca^2+^ triggered H_2_O_2_ generation, which initiated hypersensitive cell death [28]. Imidazole was used in this study to investigate the relationship between H_2_O_2_ and NO. In the incompatible combination, subsequent to the pre-injection of imidazole, NO production was inhibited (Fig 8F); in addition, the HR advancing rate decreased relative to the control (Fig 6), and the number of haustorium mother cells at the same time was higher than that in the control group (Fig 8). Therefore, we speculated that in the signal transduction pathway of HR induced by *P*. *triticina* infection, H_2_O_2_ acts at upstream of NO. It requires further study whether NO provides feedback regulation on the concentration of intracellular H_2_O_2_.
